# On the Origin of Frameshift-Robustness of the Standard Genetic Code

**DOI:** 10.1093/molbev/msab164

**Published:** 2021-05-27

**Authors:** Haiqing Xu, Jianzhi Zhang

**Affiliations:** Department of Ecology and Evolutionary Biology, University of Michigan, Ann Arbor, MI, USA

**Keywords:** mutation, mistranslation, amino acid property, natural selection, mismatch-robustness, byproduct

## Abstract

The standard genetic code (SGC) has been extensively analyzed for the biological ramifications of its nonrandom structure. For instance, mismatch errors due to point mutation or mistranslation have an overall smaller effect on the amino acid polar requirement under the SGC than under random genetic codes (RGCs). A similar observation was recently made for frameshift errors, prompting the assertion that the SGC has been shaped by natural selection for frameshift-robustness—conservation of certain amino acid properties upon a frameshift mutation or translational frameshift. However, frameshift-robustness confers no benefit because frameshifts usually create premature stop codons that cause nonsense-mediated mRNA decay or production of nonfunctional truncated proteins. We here propose that the frameshift-robustness of the SGC is a byproduct of its mismatch-robustness. Of 564 amino acid properties considered, the SGC exhibits mismatch-robustness in 93–133 properties and frameshift-robustness in 55 properties, respectively, and that the latter is largely a subset of the former. For each of the 564 real and 564 randomly constructed fake properties of amino acids, there is a positive correlation between mismatch-robustness and frameshift-robustness across one million RGCs; this correlation arises because most amino acid changes resulting from a frameshift are also achievable by a mismatch error. Importantly, the SGC does not show significantly higher frameshift-robustness in any of the 55 properties than RGCs of comparable mismatch-robustness. These findings support that the frameshift-robustness of the SGC need not originate through direct selection and can instead be a site effect of its mismatch-robustness.

## Introduction

The standard genetic code (SGC) serves as a dictionary following which the genetic information encoded in a genome is translated into proteins in almost all organisms. Ever since the unraveling of the SGC, its origin and evolution have received much attention (Pelc 1965; Sonneborn 1965; Woese 1965a; Epstein 1966; Goldberg and Wittes 1966; Pelc and Welton 1966; Wong 2005; Delarue 2007). In particular, it was noticed that the codons for the 20 amino acids are not randomly arranged in the SGC (Woese 1965b; Crick 1968). A number of authors suggested that this arrangement minimizes the impact of mismatch errors on the physicochemical properties of the encoded amino acid, where mismatch errors refer to point mutations that convert one nucleotide to another in the genome (Sonneborn 1965; Epstein 1966) or mistranslations that misrecognize one nucleotide for another during the translation of a codon (Woese 1965a; Goldberg and Wittes 1966). For example, when the amino acid property of polar requirement is considered, the SGC is worse than only one out of one million random genetic codes (RGCs) in its robustness to mismatch errors (Freeland and Hurst 1998). Subsequent studies considering stop codons and codon frequencies confirmed the extraordinary insensitivity of the SGC to mismatch errors (Gilis et al. 2001; Goodarzi et al. 2004). Although the SGC is far from being optimal in mismatch error mitigation and can be easily improved further (Archetti 2004; Novozhilov et al. 2007; Massey 2008; Santos and Monteagudo 2010; Błażej et al. 2016, 2018, 2019; Wnętrzak et al. 2018), there is no doubt that it better alleviates the deleterious effect of mismatch errors when compared with RGCs.

Recently, several groups reported that the SGC is also robust to frameshift errors (Geyer and Madany Mamlouk 2018; Wang et al. 2021; Wnętrzak et al. 2019; Bartonek et al. 2020), which can be caused by insertion/deletion mutations that alter the reading frame or translational frameshifts. Specifically, Wang et al. (2021) found that, upon a +1 or −1 frameshift, the new protein sequence tends to be similar to the original sequence when evaluated by commonly used sequence alignment scoring matrices such as BLOSUM62 (Henikoff and Henikoff 1992) and PAM250 (Dayhoff et al. 1978), as long as sites occupied by premature stop codons are skipped. Similarly, Geyer and Madany Mamlouk showed that, when the amino acid polar requirement is considered, the SGC is significantly more robust than RGCs to frameshift errors and that none of one million RGCs examined surpass the SGC in frameshift-robustness and mismatch-robustness combined (Geyer and Madany Mamlouk 2018). Instead of investigating one or a few amino acid properties, Bartonek et al. (2020) surveyed the frameshift-robustness of the SGC in 604 different properties and reached a similar conclusion that the SGC preserves a number of key physicochemical properties of amino acids upon frameshifts. These observations led to the suggestion that the SGC has been selected for frameshift-robustness during its evolution (Geyer and Madany Mamlouk 2018; Wang et al. 2021; Bartonek et al. 2020).

However, frameshift-robustness is highly unlikely to be advantageous, because frameshift errors typically cause the production of nonfunctional truncated proteins that are degraded by the cell. Furthermore, it has been suggested that gene sequences are enriched with out-of-frame stop codons to induce prompt termination of protein translation upon translational frameshifts for the benefit of minimizing the energy and resource wasted in synthesizing nonfunctional proteins (Seligmann and Pollock 2004). This ambush hypothesis is empirically supported in some prokaryotes (Tse et al. 2010; Abrahams and Hurst 2018). Furthermore, frameshifts are more likely to result in stop codons under the SGC than RGCs (Itzkovitz and Alon 2007; Kumar and Saini 2016; Wnętrzak et al. 2019). Additionally, eukaryotic mRNAs with premature stop codons in all but the last exon of intron-containing genes are degraded by the nonsense-mediated decay machinery (Nagy and Maquat 1998; Brogna and Wen 2009), minimizing the relevance of frameshift-robustness.

Considering the above facts, we propose that the SGC’s frameshift-robustness is not a result of direct natural selection. Intriguingly, it was found that, among 15 RGCs that exhibited higher mismatch-robustness than that of the SGC, almost all also showed much higher frameshift-robustness than that of average RGCs (Geyer and Madany Mamlouk 2018). This observation suggests the possibility that the SGC’s frameshift-robustness is a side effect of its mismatch-robustness. Below we provide evidence for this hypothesis and explain the mechanism through which mismatch-robustness can create frameshift-robustness.

## Results

### The SGC Is Mismatch- and Frameshift-Robust in Many Amino Acid Properties

We first attempted to uncover all amino acid properties for which the SGC is significantly more robust to mismatch or frameshift errors than RGCs. We investigated 564 amino acid properties (supplementary table S1, Supplementary Material online), including all properties considered in a previous study (Bartonek et al. 2020) except those that do not have a value for every amino acid (see Materials and Methods). We examined all pairs of sense codons that can be converted from each other by a mismatch (or frameshift) error and calculated the weighted squared difference in the particular amino acid property concerned between the corresponding amino acids, where the weight is the relative frequency of each conversion (see Materials and Methods). The smaller the weighted squared difference, referred to as the mean squared difference (MS) hereinafter, the higher the robustness of the SGC. For comparison, we simulated one million RGCs following a commonly used procedure (see Materials and Methods) and similarly measured their respective MS values. Briefly, starting from the SGC, we kept the positions of the three stop codons unchanged but randomly shuffled the identities of the 20 amino acids to generate a RGC. In theory, 20! ≈ 2.4 × 10^18^ different RGCs can be generated this way. The empirical *P* value of the robustness of the SGC is the fraction of RGCs with lower MS values than that of the SGC. We considered two distinct parameter sets to mimic mismatch errors. In the first set, which mimics generic mismatch errors (Haig and Hurst 1991), all codons have equal frequencies and all one-mismatch codon-to-codon changes have equal probabilities. In the second set, which mimics mistranslation (Freeland and Hurst 1998), we used translational errors estimated from *Escherichia coli* (Mordret et al. 2019), the only species in which such information is currently available from proteomics, to determine the relative probabilities of different codon-to-codon changes (supplementary table S2, Supplementary Material online; see Materials and Methods) whereas all codons are still assumed to have equal frequencies. The corresponding MS values are referred to as MS1 and MS2, respectively. For frameshift errors, we considered +1 (the reading frame is moved by one nucleotide toward the 3′) frameshifts and refer to the resultant MS as MS3. We did not separately consider −1 frameshifts because +1 and −1 frameshifts yield equal MS values. This is because a change from codon *i* to codon *j* by a +1 frameshift has the same probability and same effect on MS as a change from codon *j* to codon *i* by a −1 frameshift under the model considered in this study. As in previous studies (Geyer and Madany Mamlouk 2018; Wang et al. 2021; Bartonek et al. 2020), stop codons were skipped in MS3 computation.

**Fig. 1.**
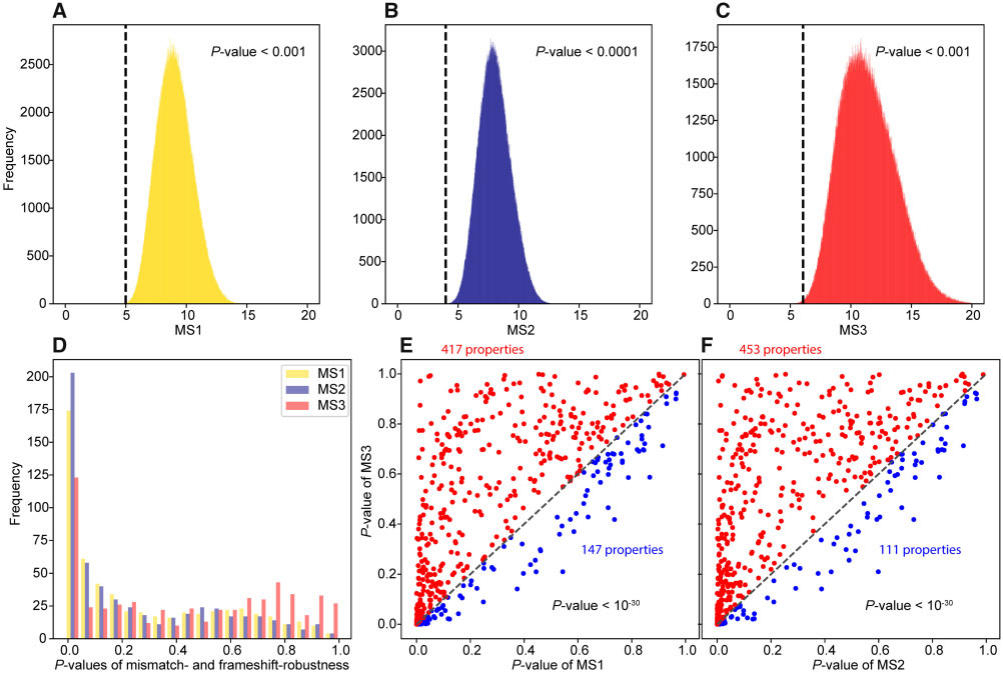
Mismatch- and frameshift-robustness of the SGC in multiple amino acid properties. (*A–C*) The frequency distribution of MS1 (*A*), MS2 (*B*), or MS3 (*C*) in polar requirement among one million RGCs. In each panel, the dashed line indicates the corresponding value for the SGC and the *P* value is the fraction of RGCs with MS values smaller than that of the SGC. (*D*) Frequency distribution of the (nominal) *P* value that measures the significance of mismatch- or frameshift-robustness of the SGC across 564 amino acid properties. The difference between the *P*-value distributions for MS1 (yellow) and MS3 (red) is significant (*P *<* *10^−11^, Kolmogorov–Smirnov test), so is the difference between the *P*-value distributions for MS2 (blue) and MS3 (red) (*P *<* *10^−15^). (*E*) The SGC’s *P* values for MS1 and MS3 in 564 amino acid properties. (*F*) The SGC’s *P* values for MS2 and MS3 in 564 amino acid properties. In (*E*) and (*F*), each dot represents one amino acid property. Dots above and below the dashed diagonal line are colored in red and blue, respectively, with their respective numbers indicated. *P* value shows the outcome of a binomial test of the equality of the numbers of red and blue dots.

We started by examining the amino acid property of polarity requirement and found that MS1 is smaller under the SGC than under 99.987% of the one million RGCs examined (fig. 1*A*). Hence, the SGC is significantly more mismatch-robust in polarity requirement than RGCs (*P *<* *10^−3^) under the first parameter set. Similar results were found for MS2 (*P *<* *10^−4^; fig. 1*B*) and MS3 (*P *<* *10^−3^; fig. 1*C*), indicating that the SGC is also significantly more mismatch-robust than RGCs under parameter set 2 and significantly more frameshift-robust than RGCs. We similarly examined 563 other amino acid properties. In total, the SGC has significantly lower MS1, MS2, and MS3 than RGCs for 93, 133, and 55 amino acid properties, respectively (supplementary table S1, Supplementary Material online), under the false discover rate (FDR) of 0.05, determined from the *P* values using the Benjamini–Hochberg method (Benjamini and Hochberg 1995).

For comparison, we simulated 564 fake amino acid properties. For each fake property, we sampled 20 random variables from the uniform distribution *U*(−1,1) to represent the property values for the 20 amino acids. We observed no property in which the SGC showed a significantly lower MS1, MS2, or MS3 value than RGCs at FDR = 0.05. Similar results were obtained when we used the normal distribution *N*(0, 1) instead of the uniform distribution. Therefore, the observed mismatch- and frameshift-robustness of the SGC in real amino acid properties is genuine.

When comparing the frequency distribution of *P* values for the SGC’s MS1, MS2, and MS3 in real amino acid properties, we noticed that MS1 and MS2 show higher incidences of low *P* values than MS3 (fig. 1*D*). Directly comparing the *P* values of MS3 with that of MS1 (fig. 1*E*) or MS2 (fig. 1*F*) revealed that, for most amino acid properties, the SGC is more robust to mismatch errors than frameshift errors. Importantly, 85% of the properties with significant frameshift-robustness also show significant mismatch-robustness measured by MS1 (supplementary table S1, Supplementary Material online), and the corresponding value is 94% when mismatch-robustness is measured by MS2 (supplementary table S1, Supplementary Material online). These large overlaps suggest the possibility that frameshift-robustness and mismatch-robustness are correlated.

### Mismatch-Robustness and Frameshift-Robustness Are Correlated across RGCs

**Fig. 2.**
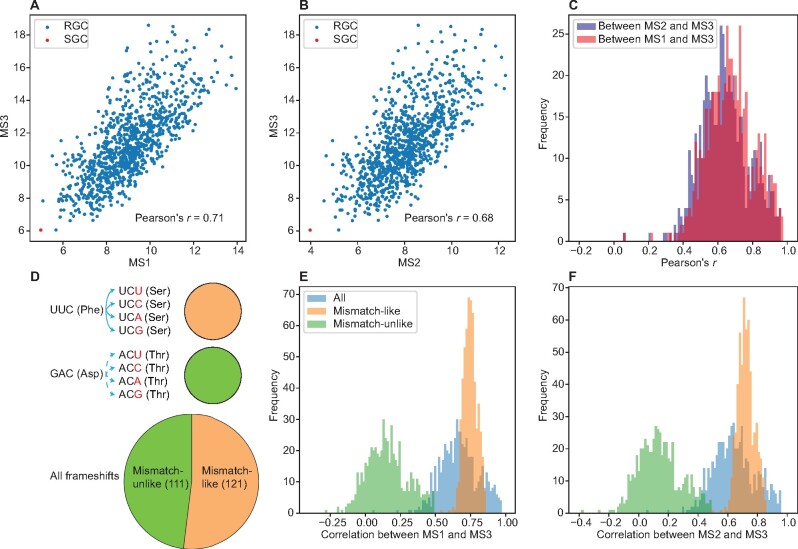
Correlation between mismatch- and frameshift-robustness and its underlying mechanism. (*A* and *B*) Pearson’s correlation (*r*) between MS1 and MS3 (*A*) or that between MS2 and MS3 (*B*) in polar requirement among one million RGCs. Each blue dot represents one RGC, and only 1,000 RGCs are shown for better viewing. The red dot represents the SGC. (*C*) Frequency distribution of the across-RGC correlation between frameshift-robustness (MS3) and mismatch-robustness (MS1 or MS2) among 564 amino acid properties. (*D*) Classification of frameshift events into two types. As examples, +1 frameshifts of codon UUC and codon GAC and their corresponding amino acids are shown. A solid line indicates a frameshift error resulting in an amino acid change that is achievable by a mismatch error, whereas a dashed line indicates a frameshift error resulting in an amino acid change that is unachievable by a mismatch error. Orange and green areas in the pie chart indicate fractions of mismatch-like and mismatch-unlike frameshifts, respectively. The number of types of frameshift events belonging to each group is given in the parentheses. (*E* and *F*) Distribution of the across-RGC correlation between frameshift-robustness (MS3) and mismatch-robustness measured by MS1 (*E*) or MS2 (*F*) among the 564 amino acid properties. MS3 is calculated based on all frameshift events (blue), mismatch-like frameshift events (orange), or mismatch-unlike frameshift events (green). The correlation between mismatch-robustness (MS1 or MS2) and mismatch-like frameshift-robustness (MS3) differs significantly from that between mismatch-robustness and mismatch-unlike frameshift-robustness (*P *<* *10^−93^, Wilcoxon signed-rank test) in both (*E*) and (*F*).

To assess the correlation between frameshift- and mismatch-robustness, we calculated Pearson’s correlation of MS3 with MS1 (fig. 2*A*) and MS2 (fig. 2*B*), respectively, among one million RGCs for the amino acid property of polar requirement. As suspected, the correlation is strongly positive in both cases (fig. 2*A* and *B*). Repeating this analysis for every one of the 564 amino acid properties, we found that the correlation between MS1 and MS3 and that between MS2 and MS3 are always positive (fig. 2*C*). This observation indicates that a positive correlation between mismatch- and frameshift-robustness does not rely on the specific amino acid property considered although the magnitude of the correlation may be. It further suggests that mismatch- and frameshift-robustness are intrinsically correlated, probably because of the relationship between the effects of mismatch and frameshift errors. Indeed, if a particular amino acid change can be achieved by both a mismatch error and a frameshift error, any variation in the physicochemical effect of the amino acid change would similarly influence MS1/2 and MS3, yielding a positive correlation between mismatch- and frameshift-robustness among different genetic codes. Let us consider the following example under the SGC, but the outcome is no different in any RGC except that the specific amino acid may vary. In a sequence of UUCG, a +1 frameshift of the codon UUC (Phe) results in the codon UCG (Ser) (fig. 2*D*). The effect of this frameshift is a Phe-to-Ser change, which can be accomplished by a single mismatch error such as UUC (Phe)-to-UCC (Ser). However, not every frameshift has an effect that can be accomplished by a mismatch. For instance, a +1 frameshift of the codon GAC (Asp) in the sequence GACU results in the codon ACU (Thr) (fig. 2*D*), but no single mismatch can convert an Asp codon to a Thr codon. Following the above logic, we divided all possible +1 frameshifts into two groups (fig. 2*D*). The mismatch-like group includes all +1 frameshifts whose effect on the encoded amino acid is accomplishable by a mismatch error. By contrast, the mismatch-unlike group includes all +1 frameshifts whose effect on the encoded amino acid is not accomplishable by a mismatch error. Among the 232 possible +1 frameshifts that do not involve stop codons (61 × 4 − 3 × 4 = 232), 121 are mismatch-like and 111 are mismatch-unlike (fig. 2*D*). This classification allows testing the hypothesis that the positive correlation between MS1/2 and MS3 is caused by the mismatch-like frameshifts but not mismatch-unlike frameshifts. Indeed, when we considered only mismatch-like frameshifts in computing MS3, the correlation between MS3 and MS1 (fig. 2*E*) or MS2 (fig. 2*F*) is strengthened. By contrast, when we considered only mismatch-unlike frameshifts in computing MS3, the correlation between MS3 and MS1 (fig. 2*E*) or MS2 (fig. 2*F*) is substantially weakened and even descends to 0 or negative for some amino acid properties.

**Fig. 3.**
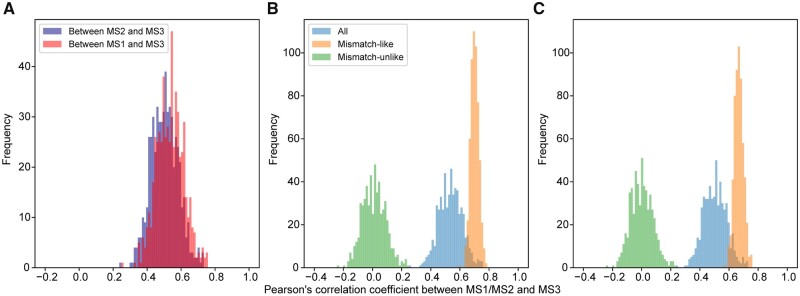
Correlation between mismatch- and frameshift-robustness in fake amino acid properties simulated using a uniform distribution. Data shown are based on 10,000 RGCs. (*A*) Frequency distribution of the across-RGC correlation between frameshift-robustness (MS3) and mismatch-robustness (MS1 or MS2) among 564 fake amino acid properties. (*B* and *C*) Frequency distribution of the across-RGC correlation between frameshift-robustness (MS3) and mismatch-robustness measured by MS1 (*B*) or MS2 (*C*) among the 564 fake amino acid properties. In (*B*) and (*C*), MS3 is calculated based on all frameshift events (blue), mismatch-like frameshift events (orange), or mismatch-unlike frameshift events (green). The correlation between mismatch-robustness (MS1 or MS2) and mismatch-like frameshift-robustness (MS3) differs significantly from that between mismatch-robustness and mismatch-unlike frameshift-robustness (*P *<* *10^−93^, Wilcoxon signed-rank test) in both (*B*) and (*C*).

If our hypothesis on the origin of the correlation between MS1/2 and MS3 among RGCs is correct, similar results as shown in figure 2*C*, *E*, and *F* should be obtained even when fake amino acid properties are examined. This is indeed the case when we analyze the 564 fake properties previously simulated under the uniform distribution (fig. 3) or normal distribution (supplementary fig. S1, Supplementary Material online). Together, these analyses demonstrate that frameshift-robustness and mismatch-robustness are intrinsically positively correlated because many frameshift errors confer the same effects on amino acid properties as mismatch errors.

### Frameshift-Robustness Is Explainable as a Byproduct of Mismatch-Robustness

**Fig. 4.**
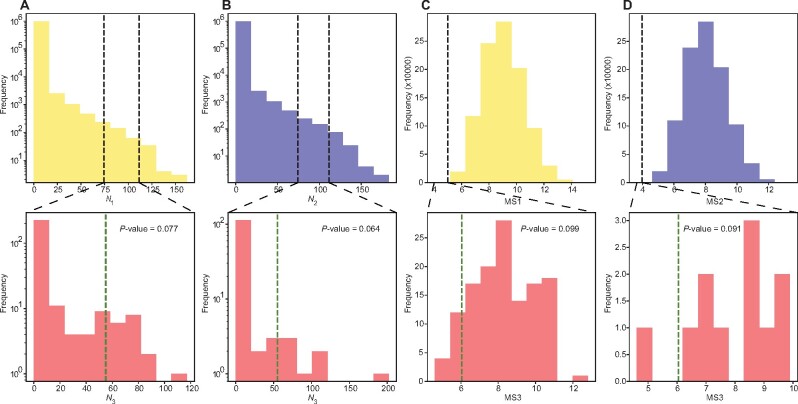
Frameshift-robustness arises as a byproduct of mismatch-robustness. (*A*) The extent of frameshift-robustness of RGCs after controlling the extent of MS1-based mismatch-robustness. The top plot shows the frequency distribution of the number (*N*_1_) of amino acid properties with significant MS1-based mismatch-robustness among one million RGCs. The black dashed lines show the 80% and 120% of the corresponding *N*_1_ of the SGC, respectively. The RGCs between the two dashed lines are control RGCs. The lower plot shows the frequency distribution of the number (*N*_3_) of amino acid properties with significant frameshift-robustness among the control RGCs, whereas the green dashed line indicates the *N*_3_ of the SGC. *P* value indicates the proportion of control RGCs that are to the right of the dashed line. (*B*) Same as (*A*) except that MS1 is replaced with MS2. (*C*) Frameshift-robustness of RGCs in polar requirement after controlling MS1-based mismatch-robustness in the same property. The top plot shows the frequency distribution of MS1 among one million RGCs. The black dashed line indicates the corresponding MS1 of the SGC. The RGCs to the left of the dashed line are control RGCs. The lower plot shows the frequency distribution of MS3 among the control RGCs, whereas the green dashed line indicates the MS3 of the SGC. *P* value indicates the proportion of control RGCs that are to the left of the dashed line. (*D*) Same as (*C*) except that MS1 is replaced with MS2.

The positive correlation between mismatch- and frameshift-robustness across one million RGCs suggests the possibility that the SGC’s frameshift-robustness arises as a byproduct of its mismatch-robustness, which, according to some authors, has resulted from natural selection for mismatch-robustness (Haig and Hurst 1991; Freeland and Hurst 1998; Koonin and Novozhilov 2009, 2017). To evaluate this possibility, for a focal RGC, we identified amino acid properties with significantly lower MS1, MS2, and MS3 at FDR = 0.05, respectively, when compared with the rest of the one million RGCs; the numbers of these properties are respectively referred to as the *N*_1_, *N*_2_, and *N*_3_ values of the focal RGC. These *N* values were obtained for each of the one million RGCs. We then picked all RGCs whose *N*_1_ is between 80% and 120% of the corresponding number (i.e., 93) of the SGC in order to have a sufficiently large sample of comparable RGCs in terms of *N*_1_ (fig. 4*A*). We found that 7.7% of these RGCs have *N*_3_ greater than that of the SGC (fig. 4*A*), suggesting that the SGC does not have an unexpectedly high *N*_3_ relative to RGCs with a comparable *N*_1_. In other words, the extent of the SGC’s frameshift-robustness measured by the number of amino acid properties with significant MS3 is explainable by its extent of mismatch-robustness. Similarly, when we picked all RGCs whose *N*_2_ is between 80% and 120% of the corresponding number (i.e., 133) of the SGC, we found that 6.4% of these RGCs have *N*_3_ greater than that of the SGC (fig. 4*B*). We repeated the above analyses by altering the range of 80–120% to 85–115%, 75–125%, or 70–130%, but found the results qualitatively unchanged.

Although the above analysis focused on the number of properties in which the SGC shows frameshift-robustness, we next examined each property in which the SGC exhibits frameshift-robustness and test if it is explainable as a byproduct of mismatch-robustness. For a given property, because the number of RGCs with a similar level of MS1 as found in the SGC is often quite small, we followed the convention of the field to examine all RGCs that have an MS1 that is equal to or smaller than that of the SGC. We then computed the fraction of these control RGCs that show an MS3 smaller than that of the SGC; this fraction is the *P* value in the test of the null hypothesis that the SGC’s MS3 is not smaller than those of the control RGCs. For example, for the property of polar requirement, *P *=* *9.9% of RGCs upon the control of MS1 have a smaller MS3 than that of the SGC (fig. 4*C*) and *P *=* *9.1% of RGCs upon the control of MS2 have a smaller MS3 than that of the SGC (fig. 4*D*). These findings suggest that for polar requirement, frameshift-robustness can be explained as a byproduct of mismatch-robustness. Using this approach, we examined all 55 properties for which the SGC shows significant frameshift-robustness. Upon the correction for multiple testing using FDR* *=* *0.05, we found that, for 54 of the 55 properties, the SGC’s frameshift-robustness (MS3) can be explained as byproducts of its mismatch-robustness (MS1 and/or MS2) (supplementary tables S3 and S4, Supplementary Material online).

The only property for which the SGC’s frameshift robustness is yet to be explained by its mismatch-robustness is “surface composition of amino acids in nuclear proteins” (ID: FUKS010104). We found that, for this property, MS3 is lower in the SGC than in each of the one million RGCs, yielding *P *=* *0. To estimate the *P* value more precisely, we simulated nine million additional RGCs. From the ten million RGCs in total, *P *=* *0.0006 after the control of MS1, which is still significant after the correction for multiple testing (adjusted *P *<* *0.05). Nevertheless, we realized that, in studying a particular property, we controlled only the MS1 or MS2 of that property, which may be insufficient because of the correlation between the mismatch-robustness in one property and the frameshift-robustness in another property (i.e., cross talks). Such cross talks are likely because the 564 properties examined are highly correlated. Indeed, cross talks can be identified when we correlate MS1 or MS2 of one property with MS3 of another property across the one million RGCs, as shown in supplementary figures S2 and S3, Supplementary Material online, for the 55 properties exhibiting frameshift-robustness in the SGC. We ranked all 564 properties by the correlation between its MS1 and the MS3 of property FUKS010104, and picked the four properties with the highest correlations. These four properties happened to be among the 55 properties with significant frameshift-robustness and the 93 properties with significant mismatch-robustness (measured by MS1) in the SGC. We selected from the ten million SGCs those that have the same MS1 as or smaller MS1 than that of the SGC for each of these four properties. From this set of control RGCs, we found that *P *=* *0.35% (adjusted *P *=* *11%) of RGCs show a smaller MS3 than that of the SGC. Thus, even for this property, frameshift-robustness is explainable by combined mismatch-robustness for several properties. Although the control of mismatch-robustness in the top four properties is somewhat arbitrary, we stress that in theory we should control mismatch-robustness in all properties, which is expected to raise the adjusted *P* further. However, such a control is not feasible because we would need many more than ten million RGCs to find a sizable set of control RGCs.

## Discussion

By surveying 564 different amino acid properties, we found that the SGC is significantly more robust to mismatch errors than RGCs for about 100 properties, greatly expanding the list of known properties for which the SGC exhibits mismatch-robustness (Haig and Hurst 1991; Freeland and Hurst 1998). In evaluating the SGC’s mismatch-robustness, we used two different sets of parameters to mimic mismatch errors, and found that both the specific amino acid properties and total number of properties showing significant mismatch-robustness vary to some extent by the parameters used. Hence, to better understand the SGC’s mismatch-robustness, we will need more precise knowledge about patterns of mismatch errors. In particular, because patterns of mismatch errors during the origin of the SGC and those of today may differ and because amino acid properties are correlated, it is possible that the properties subject to the initial (potential) selection for SGC’s mismatch-robustness may differ to some degree from those that exhibit mismatch-robustness today. Furthermore, because patterns of mismatch errors may vary among species, mismatch-robustness may also vary among species.

We found that the SGC is robust to frameshift errors in 55 amino acid properties, which overlap largely with the properties showing MS1/2-based mismatch-robustness. We observed that mismatch- and frameshift-robustness are positively correlated across one million RGCs for each of the 564 real and 564 fake properties examined. We discovered the underlying reason of this correlation—most amino acid changes caused by a frameshift is also accomplishable by a mismatch error. This is possible because of the high degeneracy of the SGC (as well as our RGCs). In particular, many frameshifts result in a new codon that differs from the original codon at two positions (e.g., UUC changes to UCG after a +1 frameshift; fig. 2*D*). However, at the level of amino acid (Phe to Ser in the above example; fig. 2*D*), the frameshift is equivalent to a single mismatch (e.g., UUC changes to UCC) because of codon degeneracy (i.e., UCG and UCC both code for Ser). Hence, the correlation between mismatch- and frameshift-robustness is ultimately attributable to the degeneracy of the genetic code. By comparing MS3 between the SGC and RGCs with comparable MS1 or MS2, we demonstrated that the SGC’s frameshift-robustness is explainable by its mismatch-robustness for each of the 55 properties. Furthermore, among the 564 properties examined, there are five factor properties that represent main variations of amino acid properties (Atchley et al. 2005). The SGC has significant frameshift-robustness in factor I and significant mismatch-robustness in factors I and IV (nominal *P *<* *0.05). Nonetheless, the frameshift-robustness in factor I is no longer significant (*P *=* *0.18) when the mismatch-robustness in factor 1 is controlled. However, as shown in supplementary tables S3 and S4, Supplementary Material online, the adjusted *P* value is rather low for most of the properties even when it is not smaller than 0.05. This is because we controlled mismatch-robustness for only one property in the analysis despite the existence of widespread cross talks (supplementary figs. S2 and S3, Supplementary Material online). As is clear from the analysis of property FUKS010104, controlling for mismatch-robustness in additional properties tends to explain better the existence of frameshift-robustness (i.e., resulting in higher adjusted *P* values). Additionally, if MS1/2 is selectively minimized under a particular scheme of mismatch error, using an arbitrary error scheme in MS1/2 computation likely underestimates the selection or the extent of mismatch-robustness. Consequently, our conclusion that the SGC’s frameshift-robustness is explainable by its mismatch-robustness is probably conservative. These findings, along with the evidence that frameshift-robustness appears useless and earlier termination of protein synthesis upon a translational frameshift is selectively favored (see Introduction), strongly support our hypothesis that the SGC’s frameshift-robustness can be a side effect of its mismatch-robustness.

In the study of either mismatch- or frameshift-robustness, it is the convention of the field to compare the SGC with RGCs generated by randomly shuffling the identities of the 20 amino acids in the code table. This comparison implies that the SGC originated through a process where one code table was selected from a sea of alternative code tables. Because each code table must have corresponding tRNAs and aminoacyl tRNA synthetases, it is highly improbable that a sea of fully formed alternative code tables coexisted and competed among themselves. It seems more likely that the SGC evolved from a more degenerate code table as new amino acids were added to life, and it is possible that the mismatch-robustness is a consequence of the coupled processes of nonrandom additions of amino acids and nonrandom reductions of code degeneracy (Wong 1975; Amirnovin 1997; Patel 2005; Wu et al. 2005; Di Giulio 2017). However, we must admit that so little is known about the initial evolution of the SGC that the above scenario is also speculative. Notwithstanding, evidence presented in this work strongly suggests that the SGC’s frameshift-robustness can be a byproduct of its mismatch-robustness regardless of the exact process that gave rise to the mismatch-robustness. Because mismatch-robustness is potentially beneficial whereas frameshift-robustness is not, the opposite hypothesis that mismatch-robustness is a byproduct of frameshift-robustness is improbable; it is also unsupported by our finding that many more amino acid properties are mismatch-robust than frameshift-robust under the SGC.

## Materials and Methods

### Amino Acid Properties

We considered all amino acid properties in a previous study (Bartonek et al. 2020) except those that do not have values for all 20 amino acids. In the end, we used 564 amino acid properties (supplementary table S1, Supplementary Material online), including 553 from the AAindex database (Kawashima et al. 2008), five factor attributes generated by factor analysis of 494 amino acid properties (Atchley et al. 2005), and six interaction preference properties of amino acids (Polyansky and Zagrovic 2013).

### Random Genetic Codes

We followed a previous study (Haig and Hurst 1991) to generate RGCs. Specifically, starting from the SGC, we kept the stop codons unchanged and shuffled the labels of the 20 amino acids for the corresponding codon sets. As a result, the block structure of synonymous codons is maintained. For example, UUC and UUU encode Phe in the SGC, and they still encode the same amino acid in each RGC, although that amino acid may not be Phe.

### Mean Squared Difference

For a given amino acid property and a specific genetic code, MS caused by mismatch or frameshift errors is calculated following previous studies (Haig and Hurst 1991; Freeland and Hurst 1998; Geyer and Madany Mamlouk 2018). Briefly, we considered all pairs of sense codons that can be converted from each other by a mismatch (or frameshift) and calculated the weighted squared difference in the property value between the corresponding amino acids using$\mathrm{MS}=\frac{\sum_{i=1}^{61}\sum_{j=1}^{61}{w_{ij}[P\left(i\right)-P\left(j\right)]}^{2}}{\sum_{i=1}^{61}\sum_{j=1}^{61}w_{ij}}$, where *i* refers to the *i*th codon, *P*(*i*)is the value of the amino acid property for codon *i*, and *w_ij_* is the relative frequency of conversion from codon *i* to *j* by a mismatch (or frameshift). We set *w_ij_* at 0 for codon pairs that cannot be converted by a mismatch (or frameshift) or codon pairs including at least one stop codon. For MS1, *w_ij_* is set at 1 for all sense codon pairs that can be converted from each other by a mismatch. For MS2, all codons have equal frequencies and the relative mismatch frequencies follow supplementary table S2, Supplementary Material online. For MS3, only +1 frameshifts are considered because the MS3 of −1 frameshifts is identical to that of +1 frameshifts.

### Estimation of Position-Specific Mistranslation Patterns

We estimated the relative frequencies of mistranslation at first (*E*_1_), second (*E*_2_), and third (*E*_3_) codon positions from *E. coli* proteomic data (Mordret et al. 2019). Briefly, the original authors inferred the mistranslation rate at each nucleotide position of each codon in a sample from peptide intensity ratios. We first averaged the intensity ratios across biological replicates. As a hypothetical example, a GTG (Val) codon at a specific location of a gene is observed to be mistranslated into Ala, Glu, and Met in various peptides. Mistranslation is mainly due to the misrecognition of a near-cognate tRNA as cognate tRNA (Mordret et al. 2019). In other words, GTG is misread as GCG (Ala), GAG (Glu), and ATG (Met), respectively. The first two cases are errors at the second codon position, whereas the third case is an error at the first codon position. We used these rates to compute the mean mistranslation rate at first (*M*_1_), second (*M*_2_), and third (*M*_3_) codon positions, respectively. We then calculated the relative frequencies as *E*_1_* *=* M*_1_/*M*_2_, *E*_2_* *=* *1, and *E*_3_* *=* M*_3_/*M*_2_. We subsequently averaged these estimates across samples excluding those from mutants or amino acid depletion media because they may not represent wild-type *E. coli* under normal environments.

From each sample, at first, second, and third codon positions, we respectively computed the mean transitional mistranslation rate and mean transversional mistranslation rate across the sample and then computed their ratio. For each codon position, we then took the median of this ratio across samples excluding those from mutants or amino acid depletion media. Because we often could not distinguish between transition and transversion errors at third codon positions, we used the average transition/transversion bias at the first two codon positions as a proxy for that at the third codon position. The estimated relative mistranslation rate at each codon position, as well as the transition/transversion rate ratio (i.e., the transition/transversion ratio multiplied by two because each nucleotide can have only one transition but two different transversions) at each codon position, are presented in supplementary table S2, Supplementary Material online, and were used in computing MS2.

## Supplementary Material

Supplementary data are available at *Molecular Biology and Evolution* online.

## Supplementary Material

msab164_Supplementary_Data
