# Supplementary material for: On the Origin of Frameshift-Robustness of the Standard Genetic Code
Source: Mol Biol Evol. 2021 May 27;38(10):4301–9. doi: 10.1093/molbev/msab164 (PMC8476161; doi:10.1093/molbev/msab164)
Supplement: msab164_Supplementary_Data [file msab164_supplementary_data.pdf]

Supplementary Materials of

"On the origin of mismatch-robustness of the standard genetic code"

H. Xu & J. Zhang

Supplementary materials include:

Tables S1-S4

Legends of supplementary figures

Figures S1-S3

Table S1. The 564 amino acid properties considered in this study, along with the references.

ID	Description	Reference	Adjusted *P* for MS1	Adjusted *P* for MS2	Adjusted *P* for MS3
Factor I	Hydrophobicity	1	0.024372	0.049128645	NS
Factor II	secondary structure	1	NS	NS	NS
Factor III	Bulkiness	1	NS	NS	NS
Factor IV	Composition	1	0.010331826	0.018475862	NS
Factor V	Charge	1	NS	NS	NS
ANDN920101	alpha-CH chemical shifts	2	NS	NS	NS
ARGP820101	Hydrophobicity index	2	NS	NS	NS
ARGP820102	Signal sequence helical potential	2	NS	NS	NS
ARGP820103	Membrane-buried preference parameters	2	NS	NS	NS
BEGF750101	Conformational parameter of inner helix	2	NS	NS	NS
BEGF750102	Conformational parameter of beta-structure	2	0.033492536	NS	NS
BEGF750103	Conformational parameter of beta-turn	2	NS	NS	NS
BHAR880101	Average flexibility indices	2	NS	NS	NS
BIGC670101	Residue volume	2	NS	NS	NS
BIOV880101	Information value for accessibility; average fraction 35%	2	0.003276571	0.006916421	0.00516624
BIOV880102	Information value for accessibility; average fraction 23%	2	0.001692	0.003321333	0.0021996
BROC820101	Retention coefficient in TFA	2	NS	NS	NS
BROC820102	Retention coefficient in HFBA	2	NS	NS	NS
BULH740101	Transfer free energy to surface	2	0.016148962	0.0412566	NS
BULH740102	Apparent partial specific volume	2	NS	NS	NS
BUNA790101	alpha-NH chemical shifts	2	NS	NS	NS
BUNA790102	alpha-CH chemical shifts	2	NS	NS	NS
BUNA790103	Spin-spin coupling constants 3JHalpha-NH	2	NS	NS	NS
BURA740101	Normalized frequency of alpha-helix	2	NS	NS	NS
BURA740102	Normalized frequency of extended structure	2	NS	NS	NS
CHAM810101	Steric parameter	2	NS	NS	NS
CHAM820101	Polarizability parameter	2	NS	NS	NS
CHAM820102	Free energy of solution in water, kcal/mole	2	NS	NS	NS
CHAM830101	The Chou-Fasman parameter of the coil conformation	2	NS	NS	NS
CHAM830102	A parameter defined from the residuals obtained from the best correlation of	2	NS	NS	NS
CHAM830103	The number of atoms in the side chain labelled 1+1	2	NS	NS	NS
CHAM830104	The number of atoms in the side chain labelled 2+1	2	NS	NS	NS

CHAM830105	The number of atoms in the side chain labelled 3+1	2	NS	NS	NS
CHAM830106	The number of bonds in the longest chain	2	NS	NS	NS
CHAM830107	A parameter of charge transfer capability	2	NS	NS	NS
CHAM830108	A parameter of charge transfer donor capability	2	NS	NS	NS
CHOC750101	Average volume of buried residue	2	NS	NS	NS
CHOC760101	Residue accessible surface area in tripeptide	2	NS	NS	NS
CHOC760102	Residue accessible surface area in folded protein	2	NS	NS	NS
CHOC760103	Proportion of residues 95% buried	2	0.033647053	NS	NS
CHOC760104	Proportion of residues 100% buried	2	NS	NS	NS
CHOP780101	Normalized frequency of beta-turn	2	NS	NS	NS
CHOP780201	Normalized frequency of alpha-helix	2	NS	NS	NS
CHOP780202	Normalized frequency of beta-sheet	2	NS	NS	NS
CHOP780203	Normalized frequency of beta-turn	2	NS	NS	NS
CHOP780204	Normalized frequency of N-terminal helix	2	NS	NS	NS
CHOP780205	Normalized frequency of C-terminal helix	2	NS	NS	NS
CHOP780206	Normalized frequency of N-terminal non helical region	2	NS	NS	NS
CHOP780207	Normalized frequency of C-terminal non helical region	2	NS	NS	NS
CHOP780208	Normalized frequency of N-terminal beta-sheet	2	0.04119929	NS	NS
CHOP780209	Normalized frequency of C-terminal beta-sheet	2	NS	NS	NS
CHOP780210	Normalized frequency of N-terminal non beta region	2	NS	NS	NS
CHOP780211	Normalized frequency of C-terminal non beta region	2	NS	NS	NS
CHOP780212	Frequency of the 1st residue in turn	2	NS	NS	NS
CHOP780213	Frequency of the 2nd residue in turn	2	NS	NS	NS
CHOP780214	Frequency of the 3rd residue in turn	2	NS	NS	NS
CHOP780215	Frequency of the 4th residue in turn	2	0.026182392	0.045052857	NS
CHOP780216	Normalized frequency of the 2nd and 3rd residues in turn	2	NS	NS	NS
CIDH920101	Normalized hydrophobicity scales for alpha-proteins	2	0.009388431	0.022074092	NS
CIDH920102	Normalized hydrophobicity scales for beta-proteins	2	0.004028571	0.010152	NS
CIDH920103	Normalized hydrophobicity scales for alpha+beta-proteins	2	0.020750242	NS	NS
CIDH920104	Normalized hydrophobicity scales for alpha/beta-proteins	2	0.00912975	0.022363826	NS
CIDH920105	Normalized average hydrophobicity scales	2	0.006778846	0.018309857	NS
COHE430101	Partial specific volume	2	NS	NS	NS
CRAJ730101	Normalized frequency of middle helix	2	NS	NS	NS
CRAJ730102	Normalized frequency of beta-sheet	2	NS	NS	NS

CRAJ730103	Normalized frequency of turn	2	NS	NS	NS
DAWD720101	Size	2	NS	NS	NS
DAYM780101	Amino acid composition	2	NS	NS	NS
DAYM780201	Relative mutability	2	NS	NS	NS
DESM900101	Membrane preference for cytochrome b: MPH89	2	0.008687419	0.022122269	0.008580857
DESM900102	Average membrane preference: AMP07	2	0.010627371	0.022074092	0.0147768
EISD840101	Consensus normalized hydrophobicity scale	2	NS	NS	NS
EISD860101	Solvation free energy	2	NS	NS	NS
EISD860102	Atom-based hydrophobic moment	2	NS	NS	NS
EISD860103	Direction of hydrophobic moment	2	0.003118588	0.0073555	NS
FASG760101	Molecular weight	2	NS	NS	NS
FASG760102	Melting point	2	NS	NS	NS
FASG760103	Optical rotation	2	NS	NS	NS
FASG760104	pK-N	2	NS	NS	NS
FASG760105	pK-C	2	NS	NS	NS
FAUJ830101	Hydrophobic parameter pi	2	0.0060912	0.017559923	0.0101144
FAUJ880101	Graph shape index	2	NS	NS	NS
FAUJ880102	Smoothed upsilon steric parameter	2	NS	NS	NS
FAUJ880103	Normalized van der Waals volume	2	NS	NS	NS
FAUJ880104	STERIMOL length of the side chain	2	NS	NS	NS
FAUJ880105	STERIMOL minimum width of the side chain	2	NS	NS	NS
FAUJ880106	STERIMOL maximum width of the side chain	2	NS	NS	NS
FAUJ880107	N.m.r. chemical shift of alpha-carbon	2	NS	NS	NS
FAUJ880108	Localized electrical effect	2	NS	NS	NS
FAUJ880109	Number of hydrogen bond donors	2	NS	NS	NS
FAUJ880110	Number of full nonbonding orbitals	2	NS	NS	NS
FAUJ880111	Positive charge	2	NS	NS	NS
FAUJ880112	Negative charge	2	NS	NS	NS
FAUJ880113	pK-a	2	NS	NS	NS
FINA770101	Helix-coil equilibrium constant	2	NS	NS	NS
FINA910101	Helix initiation parameter at posision i-1	2	NS	NS	NS
FINA910102	Helix initiation parameter at posision i,i+1,i+2	2	NS	NS	NS
FINA910103	Helix termination parameter at posision j-2,j-1,j	2	NS	NS	NS
FINA910104	Helix termination parameter at posision j+1	2	NS	NS	NS

GARJ730101	Partition coefficient	2	0.035541479	0.04711618	NS
GEIM800101	Alpha-helix indices	2	NS	NS	NS
GEIM800102	Alpha-helix indices for alpha-proteins	2	NS	NS	NS
GEIM800103	Alpha-helix indices for beta-proteins	2	NS	NS	NS
GEIM800104	Alpha-helix indices for alpha/beta-proteins	2	NS	NS	NS
GEIM800105	Beta-strand indices	2	NS	NS	NS
GEIM800106	Beta-strand indices for beta-proteins	2	NS	NS	NS
GEIM800107	Beta-strand indices for alpha/beta-proteins	2	NS	NS	NS
GEIM800108	Aperiodic indices	2	NS	NS	NS
GEIM800109	Aperiodic indices for alpha-proteins	2	NS	NS	NS
GEIM800110	Aperiodic indices for beta-proteins	2	NS	NS	NS
GEIM800111	Aperiodic indices for alpha/beta-proteins	2	NS	NS	NS
GOLD730101	Hydrophobicity factor	2	NS	NS	NS
GOLD730102	Residue volume	2	NS	NS	NS
GRAR740101	Composition	2	NS	NS	NS
GRAR740102	Polarity	2	0.00141	0.003321333	0.002397
GRAR740103	Volume	2	NS	NS	NS
GUYH850101	Partition energy	2	NS	NS	NS
HOPA770101	Hydration number	2	0.01388192	0.037452456	NS
HOPT810101	Hydrophilicity value	2	0.044090476	NS	0.0147768
HUTJ700101	Heat capacity	2	NS	NS	NS
HUTJ700102	Absolute entropy	2	NS	NS	NS
HUTJ700103	Entropy of formation	2	NS	NS	NS
ISOY800101	Normalized relative frequency of alpha-helix	2	NS	NS	NS
ISOY800102	Normalized relative frequency of extended structure	2	NS	NS	NS
ISOY800103	Normalized relative frequency of bend	2	NS	NS	NS
ISOY800104	Normalized relative frequency of bend R	2	NS	NS	NS
ISOY800105	Normalized relative frequency of bend S	2	NS	NS	NS
ISOY800106	Normalized relative frequency of helix end	2	NS	NS	NS
ISOY800107	Normalized relative frequency of double bend	2	NS	NS	NS
ISOY800108	Normalized relative frequency of coil	2	NS	NS	NS
JANJ780101	Average accessible surface area	2	NS	NS	NS
JANJ780102	Percentage of buried residues	2	NS	NS	NS
JANJ780103	Percentage of exposed residues	2	NS	NS	NS

JANJ790101	Ratio of buried and accessible molar fractions	2	NS	NS	NS
JANJ790102	Transfer free energy	2	NS	NS	NS
JOND750101	Hydrophobicity	2	NS	NS	NS
JOND750102	pK	2	NS	NS	NS
JOND920101	Relative frequency of occurrence	2	NS	NS	NS
JOND920102	Relative mutability	2	NS	NS	NS
JUKT750101	Amino acid distribution	2	NS	NS	NS
JUNJ780101	Sequence frequency	2	NS	NS	NS
KANM800101	Average relative probability of helix	2	NS	NS	NS
KANM800102	Average relative probability of beta-sheet	2	NS	NS	NS
KANM800103	Average relative probability of inner helix	2	NS	NS	NS
KANM800104	Average relative probability of inner beta-sheet	2	NS	NS	NS
KARP850101	Flexibility parameter for no rigid neighbors	2	NS	NS	NS
KARP850102	Flexibility parameter for one rigid neighbor	2	0.019784864	0.04711618	0.0147768
KARP850103	Flexibility parameter for two rigid neighbors	2	0.044840186	NS	0.011844
KHAG800101	The Kerr-constant increments	2	NS	NS	NS
KLEP840101	Net charge	2	NS	NS	NS
KRIW710101	Side chain interaction parameter	2	NS	NS	0.019896
KRIW790101	Side chain interaction parameter	2	0.00354192	0.006916421	0.0021996
KRIW790102	Fraction of site occupied by water	2	0.012133833	0.018309857	0.0021996
KRIW790103	Side chain volume	2	NS	NS	NS
KYTJ820101	Hydropathy index	2	0.015960468	0.048467217	NS
LAWE840101	Transfer free energy, CHP/water	2	NS	NS	NS
LEVM760101	Hydrophobic parameter	2	NS	NS	0.042012679
LEVM760102	Distance between C-alpha and centroid of side chain	2	NS	NS	NS
LEVM760103	Side chain angle theta	2	NS	NS	NS
LEVM760104	Side chain torsion angle phi	2	NS	NS	NS
LEVM760105	Radius of gyration of side chain	2	NS	NS	NS
LEVM760106	van der Waals parameter R0	2	NS	NS	NS
LEVM760107	van der Waals parameter epsilon	2	NS	NS	NS
LEVM780101	Normalized frequency of alpha-helix, with weights	2	NS	NS	NS
LEVM780102	Normalized frequency of beta-sheet, with weights	2	NS	NS	NS
LEVM780103	Normalized frequency of reverse turn, with weights	2	NS	NS	NS
LEVM780104	Normalized frequency of alpha-helix, unweighted	2	NS	NS	NS

LEVM780105	Normalized frequency of beta-sheet, unweighted	2	NS	NS	NS
LEVM780106	Normalized frequency of reverse turn, unweighted	2	NS	NS	NS
LEWP710101	Frequency of occurrence in beta-bends	2	NS	NS	NS
LIFS790101	Conformational preference for all beta-strands	2	NS	NS	NS
LIFS790102	Conformational preference for parallel beta-strands	2	NS	NS	NS
LIFS790103	Conformational preference for antiparallel beta-strands	2	NS	NS	NS
MANP780101	Average surrounding hydrophobicity	2	0.037969785	NS	NS
MAXF760101	Normalized frequency of alpha-helix	2	NS	NS	NS
MAXF760102	Normalized frequency of extended structure	2	NS	NS	NS
MAXF760103	Normalized frequency of zeta R	2	NS	NS	NS
MAXF760104	Normalized frequency of left-handed alpha-helix	2	NS	NS	NS
MAXF760105	Normalized frequency of zeta L	2	NS	NS	NS
MAXF760106	Normalized frequency of alpha region	2	NS	NS	NS
MCMT640101	Refractivity	2	0.035541479	NS	NS
MEEJ800101	Retention coefficient in HPLC, pH7.4	2	0.005787714	0.009044143	NS
MEEJ800102	Retention coefficient in HPLC, pH2.1	2	0.026182392	0.046948138	NS
MEEJ810101	Retention coefficient in NaClO4	2	0.005909182	0.0140013	NS
MEEJ810102	Retention coefficient in NaH2PO4	2	0.010331826	0.022074092	NS
MEIH800101	Average reduced distance for C-alpha	2	0.0063215	0.017856453	NS
MEIH800102	Average reduced distance for side chain	2	0.004550897	0.010152	0.018007714
MEIH800103	Average side chain orientation angle	2	0.001692	0.003321333	0.0041736
MIYS850101	Effective partition energy	2	0.001692	0.003321333	0.018007714
NAGK730101	Normalized frequency of alpha-helix	2	NS	NS	NS
NAGK730102	Normalized frequency of bata-structure	2	NS	NS	NS
NAGK730103	Normalized frequency of coil	2	NS	NS	NS
NAKH900101	AA composition of total proteins	2	NS	NS	NS
NAKH900102	SD of AA composition of total proteins	2	NS	NS	NS
NAKH900103	AA composition of mt-proteins	2	NS	NS	NS
NAKH900104	Normalized composition of mt-proteins	2	0.047636308	NS	NS
NAKH900105	AA composition of mt-proteins from animal	2	NS	NS	NS
NAKH900106	Normalized composition from animal	2	NS	NS	NS
NAKH900107	AA composition of mt-proteins from fungi and plant	2	NS	NS	NS
NAKH900108	Normalized composition from fungi and plant	2	0.044840186	0.045052857	0.018297209
NAKH900109	AA composition of membrane proteins	2	NS	NS	NS

NAKH900110	Normalized composition of membrane proteins	2	0.035541479	NS	NS
NAKH900111	Transmembrane regions of non-mt-proteins	2	NS	NS	NS
NAKH900112	Transmembrane regions of mt-proteins	2	NS	NS	NS
NAKH900113	Ratio of average and computed composition	2	NS	NS	NS
NAKH920101	AA composition of CYT of single-spanning proteins	2	NS	NS	NS
NAKH920102	AA composition of CYT2 of single-spanning proteins	2	NS	NS	NS
NAKH920103	AA composition of EXT of single-spanning proteins	2	NS	NS	NS
NAKH920104	AA composition of EXT2 of single-spanning proteins	2	NS	NS	NS
NAKH920105	AA composition of MEM of single-spanning proteins	2	NS	NS	NS
NAKH920106	AA composition of CYT of multi-spanning proteins	2	NS	NS	NS
NAKH920107	AA composition of EXT of multi-spanning proteins	2	NS	NS	NS
NAKH920108	AA composition of MEM of multi-spanning proteins	2	NS	NS	NS
NISK800101	8 A contact number	2	0.016592741	NS	0.046571778
NISK860101	14 A contact number	2	0.003276571	0.007592308	0.03105525
NOZY710101	Transfer energy, organic solvent/water	2	0.006132	0.012456343	0.035189176
OOBM770101	Average non-bonded energy per atom	2	0.033647053	NS	NS
OOBM770102	Short and medium range non-bonded energy per atom	2	NS	NS	NS
OOBM770103	Long range non-bonded energy per atom	2	0	0.001692	0.003791333
OOBM770104	Average non-bonded energy per residue	2	0.035541479	NS	NS
OOBM770105	Short and medium range non-bonded energy per residue	2	NS	NS	NS
OOBM850101	Optimized beta-structure-coil equilibrium constant	2	NS	NS	NS
OOBM850102	Optimized propensity to form reverse turn	2	0.01388192	0.020794033	NS
OOBM850103	Optimized transfer energy parameter	2	NS	NS	NS
OOBM850104	Optimized average non-bonded energy per atom	2	0.005584976	0.0073555	NS
OOBM850105	Optimized side chain interaction parameter	2	NS	NS	NS
PALJ810101	Normalized frequency of alpha-helix from LG	2	NS	NS	NS
PALJ810102	Normalized frequency of alpha-helix from CF	2	NS	NS	NS
PALJ810103	Normalized frequency of beta-sheet from LG	2	NS	NS	NS
PALJ810104	Normalized frequency of beta-sheet from CF	2	NS	NS	NS
PALJ810105	Normalized frequency of turn from LG	2	NS	NS	NS
PALJ810106	Normalized frequency of turn from CF	2	NS	NS	NS
PALJ810107	Normalized frequency of alpha-helix in all-alpha class	2	NS	NS	NS
PALJ810108	Normalized frequency of alpha-helix in alpha+beta class	2	NS	NS	NS
PALJ810109	Normalized frequency of alpha-helix in alpha/beta class	2	NS	NS	NS

PALJ810110	Normalized frequency of beta-sheet in all-beta class	2	NS	NS	NS
PALJ810111	Normalized frequency of beta-sheet in alpha+beta class	2	NS	NS	NS
PALJ810112	Normalized frequency of beta-sheet in alpha/beta class	2	NS	NS	NS
PALJ810113	Normalized frequency of turn in all-alpha class	2	NS	NS	NS
PALJ810114	Normalized frequency of turn in all-beta class	2	0.032778655	NS	NS
PALJ810115	Normalized frequency of turn in alpha+beta class	2	NS	NS	NS
PALJ810116	Normalized frequency of turn in alpha/beta class	2	NS	NS	NS
PARJ860101	HPLC parameter	2	0.005076	0.013695077	NS
PLIV810101	Partition coefficient	2	0.0082156	0.027322667	NS
PONP800101	Surrounding hydrophobicity in folded form	2	0.020177258	NS	NS
PONP800102	Average gain in surrounding hydrophobicity	2	0.007767368	0.022363826	0.018881182
PONP800103	Average gain ratio in surrounding hydrophobicity	2	0.0018048	0.0049256	0.0021996
PONP800104	Surrounding hydrophobicity in alpha-helix	2	NS	NS	NS
PONP800105	Surrounding hydrophobicity in beta-sheet	2	NS	NS	NS
PONP800106	Surrounding hydrophobicity in turn	2	0.010331826	0.018309857	NS
PONP800107	Accessibility reduction ratio	2	0.018824329	0.047258242	NS
PONP800108	Average number of surrounding residues	2	0.01617975	0.045052857	NS
PRAM820101	Intercept in regression analysis	2	NS	NS	NS
PRAM820102	Slope in regression analysis x 1.0E1	2	NS	NS	NS
PRAM820103	Correlation coefficient in regression analysis	2	NS	NS	NS
PRAM900101	Hydrophobicity	2	NS	NS	NS
PRAM900102	Relative frequency in alpha-helix	2	NS	NS	NS
PRAM900103	Relative frequency in beta-sheet	2	NS	NS	NS
PRAM900104	Relative frequency in reverse-turn	2	NS	NS	NS
PTIO830101	Helix-coil equilibrium constant	2	NS	NS	NS
PTIO830102	Beta-coil equilibrium constant	2	NS	NS	NS
QIAN880101	Weights for alpha-helix at the window position of -6	2	NS	NS	NS
QIAN880102	Weights for alpha-helix at the window position of -5	2	NS	NS	NS
QIAN880103	Weights for alpha-helix at the window position of -4	2	NS	NS	NS
QIAN880104	Weights for alpha-helix at the window position of -3	2	NS	NS	NS
QIAN880105	Weights for alpha-helix at the window position of -2	2	NS	NS	NS
QIAN880106	Weights for alpha-helix at the window position of -1	2	NS	NS	NS
QIAN880107	Weights for alpha-helix at the window position of 0	2	NS	NS	NS
QIAN880108	Weights for alpha-helix at the window position of 1	2	NS	NS	NS

QIAN880109	Weights for alpha-helix at the window position of 2	2	NS	NS	NS
QIAN880110	Weights for alpha-helix at the window position of 3	2	NS	NS	NS
QIAN880111	Weights for alpha-helix at the window position of 4	2	NS	NS	NS
QIAN880112	Weights for alpha-helix at the window position of 5	2	NS	NS	NS
QIAN880113	Weights for alpha-helix at the window position of 6	2	NS	NS	NS
QIAN880114	Weights for beta-sheet at the window position of -6	2	NS	NS	NS
QIAN880115	Weights for beta-sheet at the window position of -5	2	NS	NS	NS
QIAN880116	Weights for beta-sheet at the window position of -4	2	NS	NS	NS
QIAN880117	Weights for beta-sheet at the window position of -3	2	NS	NS	NS
QIAN880118	Weights for beta-sheet at the window position of -2	2	0.049598075	NS	NS
QIAN880119	Weights for beta-sheet at the window position of -1	2	NS	NS	NS
QIAN880120	Weights for beta-sheet at the window position of 0	2	NS	NS	NS
QIAN880121	Weights for beta-sheet at the window position of 1	2	NS	NS	NS
QIAN880122	Weights for beta-sheet at the window position of 2	2	NS	NS	NS
QIAN880123	Weights for beta-sheet at the window position of 3	2	NS	NS	NS
QIAN880124	Weights for beta-sheet at the window position of 4	2	NS	NS	NS
QIAN880125	Weights for beta-sheet at the window position of 5	2	NS	NS	NS
QIAN880126	Weights for beta-sheet at the window position of 6	2	NS	NS	NS
QIAN880127	Weights for coil at the window position of -6	2	NS	NS	NS
QIAN880128	Weights for coil at the window position of -5	2	NS	NS	NS
QIAN880129	Weights for coil at the window position of -4	2	NS	NS	NS
QIAN880130	Weights for coil at the window position of -3	2	0.04119929	NS	NS
QIAN880131	Weights for coil at the window position of -2	2	NS	NS	NS
QIAN880132	Weights for coil at the window position of -1	2	NS	NS	NS
QIAN880133	Weights for coil at the window position of 0	2	NS	NS	NS
QIAN880134	Weights for coil at the window position of 1	2	NS	NS	NS
QIAN880135	Weights for coil at the window position of 2	2	NS	NS	NS
QIAN880136	Weights for coil at the window position of 3	2	NS	NS	NS
QIAN880137	Weights for coil at the window position of 4	2	NS	NS	NS
QIAN880138	Weights for coil at the window position of 5	2	NS	NS	NS
QIAN880139	Weights for coil at the window position of 6	2	NS	NS	NS
RACS770101	Average reduced distance for C-alpha	2	0.020750242	NS	NS
RACS770102	Average reduced distance for side chain	2	0.006778846	0.017559923	NS
RACS770103	Side chain orientational preference	2	0.006778846	0.012878	0.003888632

RACS820101	Average relative fractional occurrence in A0	2	NS	NS	NS
RACS820102	Average relative fractional occurrence in AR	2	NS	NS	NS
RACS820103	Average relative fractional occurrence in AL	2	NS	NS	0.039013615
RACS820104	Average relative fractional occurrence in EL	2	NS	NS	NS
RACS820105	Average relative fractional occurrence in E0	2	0.031052229	NS	0.031802694
RACS820106	Average relative fractional occurrence in ER	2	NS	NS	NS
RACS820107	Average relative fractional occurrence in A0	2	NS	NS	NS
RACS820108	Average relative fractional occurrence in AR	2	NS	NS	NS
RACS820109	Average relative fractional occurrence in AL	2	NS	NS	NS
RACS820110	Average relative fractional occurrence in EL	2	NS	NS	NS
RACS820111	Average relative fractional occurrence in E0	2	NS	NS	NS
RACS820112	Average relative fractional occurrence in ER	2	NS	NS	NS
RACS820113	Value of theta	2	NS	NS	NS
RACS820114	Value of theta	2	NS	NS	NS
RADA880101	Transfer free energy from chx to wat	2	NS	NS	NS
RADA880102	Transfer free energy from oct to wat	2	NS	NS	NS
RADA880103	Transfer free energy from vap to chx	2	NS	NS	NS
RADA880104	Transfer free energy from chx to oct	2	NS	NS	NS
RADA880105	Transfer free energy from vap to oct	2	NS	NS	NS
RADA880106	Accessible surface area	2	NS	NS	NS
RADA880107	Energy transfer from out to in	2	NS	NS	NS
RADA880108	Mean polarity	2	0.005584976	0.013695077	0.0101144
RICJ880101	Relative preference value at N"	2	NS	NS	NS
RICJ880102	Relative preference value at N'	2	NS	NS	NS
RICJ880103	Relative preference value at N-cap	2	NS	NS	NS
RICJ880104	Relative preference value at N1	2	NS	NS	NS
RICJ880105	Relative preference value at N2	2	NS	NS	NS
RICJ880106	Relative preference value at N3	2	NS	NS	NS
RICJ880107	Relative preference value at N4	2	NS	NS	NS
RICJ880108	Relative preference value at N5	2	NS	NS	NS
RICJ880109	Relative preference value at Mid	2	NS	NS	NS
RICJ880110	Relative preference value at C5	2	NS	NS	NS
RICJ880111	Relative preference value at C4	2	NS	NS	NS
RICJ880112	Relative preference value at C3	2	NS	NS	NS

RICJ880113	Relative preference value at C2	2	NS	NS	NS
RICJ880114	Relative preference value at C1	2	NS	NS	NS
RICJ880115	Relative preference value at C-cap	2	NS	NS	NS
RICJ880116	Relative preference value at C'	2	NS	NS	NS
RICJ880117	Relative preference value at C"	2	NS	NS	NS
ROBB760101	Information measure for alpha-helix	2	NS	NS	NS
ROBB760102	Information measure for N-terminal helix	2	NS	NS	NS
ROBB760103	Information measure for middle helix	2	NS	NS	NS
ROBB760104	Information measure for C-terminal helix	2	NS	NS	NS
ROBB760105	Information measure for extended	2	NS	NS	NS
ROBB760106	Information measure for pleated-sheet	2	NS	NS	NS
ROBB760107	Information measure for extended without H-bond	2	NS	NS	NS
ROBB760108	Information measure for turn	2	NS	NS	NS
ROBB760109	Information measure for N-terminal turn	2	NS	NS	NS
ROBB760110	Information measure for middle turn	2	0.02806464	NS	NS
ROBB760111	Information measure for C-terminal turn	2	NS	NS	NS
ROBB760112	Information measure for coil	2	NS	NS	NS
ROBB760113	Information measure for loop	2	NS	NS	NS
ROBB790101	Hydration free energy	2	0.018591614	0.037326545	NS
ROSG850101	Mean area buried on transfer	2	0.031547444	NS	NS
ROSG850102	Mean fractional area loss	2	0.0034075	0.0073555	0.002397
ROSM880101	Side chain hydropathy, uncorrected for solvation	2	NS	NS	NS
ROSM880102	Side chain hydropathy, corrected for solvation	2	NS	NS	NS
ROSM880103	Loss of Side chain hydropathy by helix formation	2	NS	NS	NS
SIMZ760101	Transfer free energy	2	NS	NS	NS
SNEP660101	Principal component I	2	NS	NS	NS
SNEP660102	Principal component II	2	NS	NS	NS
SNEP660103	Principal component III	2	0.049264545	NS	NS
SNEP660104	Principal component IV	2	NS	NS	NS
SUEM840101	Zimm-Bragg parameter s at 20 C	2	NS	NS	NS
SUEM840102	Zimm-Bragg parameter sigma x 1.0E4	2	NS	NS	NS
SWER830101	Optimal matching hydrophobicity	2	0.001692	0.004841	0.010416375
TANS770101	Normalized frequency of alpha-helix	2	NS	NS	NS
TANS770102	Normalized frequency of isolated helix	2	NS	NS	NS

TANS770103	Normalized frequency of extended structure	2	NS	NS	NS
TANS770104	Normalized frequency of chain reversal R	2	NS	NS	NS
TANS770105	Normalized frequency of chain reversal S	2	NS	NS	NS
TANS770106	Normalized frequency of chain reversal D	2	NS	NS	NS
TANS770107	Normalized frequency of left-handed helix	2	NS	NS	NS
TANS770108	Normalized frequency of zeta R	2	NS	NS	NS
TANS770109	Normalized frequency of coil	2	NS	NS	NS
TANS770110	Normalized frequency of chain reversal	2	NS	NS	NS
VASM830101	Relative population of conformational state A	2	NS	NS	NS
VASM830102	Relative population of conformational state C	2	NS	NS	NS
VASM830103	Relative population of conformational state E	2	NS	NS	NS
VELV850101	Electron-ion interaction potential	2	NS	NS	NS
VENT840101	Bitterness	2	0.044840186	NS	NS
VHEG790101	Transfer free energy to lipophilic phase	2	NS	NS	NS
WARP780101	Average interactions per side chain atom	2	0.014723368	0.036259263	NS
WEBA780101	RF value in high salt chromatography	2	0.018824329	0.023567143	NS
WERD780101	Propensity to be buried inside	2	0.001692	0.003321333	0.0021996
WERD780102	Free energy change of epsilon	2	NS	NS	NS
WERD780103	Free energy change of alpha	2	NS	NS	NS
WERD780104	Free energy change of epsilon	2	0.029662019	0.043971111	NS
WOEC730101	Polar requirement	2	0.001692	0.0049256	0.010596364
WOLR810101	Hydration potential	2	NS	NS	NS
WOLS870101	Principal property value z1	2	0.004028571	0.012092824	NS
WOLS870102	Principal property value z2	2	NS	NS	NS
WOLS870103	Principal property value z3	2	NS	NS	NS
YUTK870101	Unfolding Gibbs energy in water, pH7.0	2	NS	NS	NS
YUTK870102	Unfolding Gibbs energy in water, pH9.0	2	NS	NS	NS
YUTK870103	Activation Gibbs energy of unfolding, pH7.0	2	NS	NS	NS
YUTK870104	Activation Gibbs energy of unfolding, pH9.0	2	NS	NS	NS
ZASB820101	Dependence of partition coefficient on ionic strength	2	0.003276571	0.006916421	NS
ZIMJ680101	Hydrophobicity	2	NS	NS	NS
ZIMJ680102	Bulkiness	2	NS	NS	NS
ZIMJ680103	Polarity	2	NS	NS	NS
ZIMJ680104	Isoelectric point	2	NS	NS	NS

ZIMJ680105	RF rank	2	0.006132	0.018475862	NS
AURR980101	Normalized positional residue frequency at helix termini N4'	2	NS	NS	NS
AURR980102	Normalized positional residue frequency at helix termini N"'	2	NS	NS	NS
AURR980103	Normalized positional residue frequency at helix termini N"	2	NS	NS	NS
AURR980104	Normalized positional residue frequency at helix termini N'	2	NS	NS	NS
AURR980105	Normalized positional residue frequency at helix termini Nc	2	NS	NS	NS
AURR980106	Normalized positional residue frequency at helix termini N1	2	NS	NS	NS
AURR980107	Normalized positional residue frequency at helix termini N2	2	NS	NS	NS
AURR980108	Normalized positional residue frequency at helix termini N3	2	NS	NS	NS
AURR980109	Normalized positional residue frequency at helix termini N4	2	NS	NS	NS
AURR980110	Normalized positional residue frequency at helix termini N5	2	NS	NS	NS
AURR980111	Normalized positional residue frequency at helix termini C5	2	NS	NS	NS
AURR980112	Normalized positional residue frequency at helix termini C4	2	NS	NS	NS
AURR980113	Normalized positional residue frequency at helix termini C3	2	NS	NS	NS
AURR980114	Normalized positional residue frequency at helix termini C2	2	NS	NS	NS
AURR980115	Normalized positional residue frequency at helix termini C1	2	NS	NS	NS
AURR980116	Normalized positional residue frequency at helix termini Cc	2	NS	NS	NS
AURR980117	Normalized positional residue frequency at helix termini C'	2	NS	NS	NS
AURR980118	Normalized positional residue frequency at helix termini C"	2	NS	NS	NS
AURR980119	Normalized positional residue frequency at helix termini C"'	2	NS	NS	NS
AURR980120	Normalized positional residue frequency at helix termini C4'	2	NS	NS	NS
ONEK900101	Delta G values for the peptides extrapolated to 0 M urea	2	NS	NS	NS
ONEK900102	Helix formation parameters	2	NS	NS	NS
VINM940101	Normalized flexibility parameters	2	0.002256	0.007592308	0.002417143
VINM940102	Normalized flexibility parameters	2	0.009041905	0.037452456	NS
VINM940103	Normalized flexibility parameters	2	0.007886441	0.017559923	0.0021996
VINM940104	Normalized flexibility parameters	2	NS	NS	0.048883418
MUNV940101	Free energy in alpha-helical conformation	2	NS	NS	NS
MUNV940102	Free energy in alpha-helical region	2	NS	NS	NS
MUNV940103	Free energy in beta-strand conformation	2	NS	NS	NS
MUNV940104	Free energy in beta-strand region	2	NS	NS	NS
MUNV940105	Free energy in beta-strand region	2	NS	NS	NS
WIMW960101	Free energies of transfer of AcWl-X-LL peptides from bilayer interface to	2	0.001692	0.003321333	0.003791333
KIMC930101	Thermodynamic beta sheet propensity	2	NS	NS	NS

MONM990101	Turn propensity scale for transmembrane helices	2	0.031547444	NS	NS
BLAM930101	Alpha helix propensity of position 44 in T4 lysozyme	2	NS	NS	NS
PARS000101	p-Values of mesophilic proteins based on the distributions of B values	2	0.016148962	NS	NS
PARS000102	p-Values of thermophilic proteins based on the distributions of B values	2	0.007342071	0.022074092	0.019896
KUMS000101	Distribution of amino acid residues in the 18 non-redundant families of	2	0.042173664	NS	NS
KUMS000102	Distribution of amino acid residues in the 18 non-redundant families of	2	NS	NS	NS
KUMS000103	Distribution of amino acid residues in the alpha-helices in thermophilic	2	NS	NS	NS
KUMS000104	Distribution of amino acid residues in the alpha-helices in mesophilic	2	NS	NS	NS
TAKK010101	Side-chain contribution to protein stability	2	NS	NS	NS
FODM020101	Propensity of amino acids within pi-helices	2	NS	NS	NS
NADH010101	Hydropathy scale based on self-information values in the two-state model	2	NS	NS	NS
NADH010102	Hydropathy scale based on self-information values in the two-state model	2	0.007342071	0.017559923	0.0147768
NADH010103	Hydropathy scale based on self-information values in the two-state model	2	0.005584976	0.012092824	0.00516624
NADH010104	Hydropathy scale based on self-information values in the two-state model	2	0.005584976	0.012911027	0.00516624
NADH010105	Hydropathy scale based on self-information values in the two-state model	2	0.022682609	0.046838233	NS
NADH010106	Hydropathy scale based on self-information values in the two-state model	2	NS	NS	NS
NADH010107	Hydropathy scale based on self-information values in the two-state model	2	NS	NS	NS
MONM990201	Averaged turn propensities in a transmembrane helix	2	0.029662019	NS	NS
KOEP990101	Alpha-helix propensity derived from designed sequences	2	NS	NS	NS
KOEP990102	Beta-sheet propensity derived from designed sequences	2	NS	NS	NS
CEDJ970101	Composition of amino acids in extracellular proteins	2	NS	NS	NS
CEDJ970102	Composition of amino acids in anchored proteins	2	NS	NS	NS
CEDJ970103	Composition of amino acids in membrane proteins	2	NS	NS	NS
CEDJ970104	Composition of amino acids in intracellular proteins	2	NS	NS	NS
CEDJ970105	Composition of amino acids in nuclear proteins	2	NS	NS	NS
FUKS010101	Surface composition of amino acids in intracellular proteins of thermophiles	2	0.049264545	NS	0.011924571
FUKS010102	Surface composition of amino acids in intracellular proteins of mesophiles	2	0.001692	0.003321333	0.00141
FUKS010103	Surface composition of amino acids in extracellular proteins of mesophiles	2	0.019784864	0.046838233	NS
FUKS010104	Surface composition of amino acids in nuclear proteins	2	0.005584976	0.012092824	0
FUKS010105	Interior composition of amino acids in intracellular proteins of thermophiles	2	NS	NS	NS
FUKS010106	Interior composition of amino acids in intracellular proteins of mesophiles	2	NS	NS	NS
FUKS010107	Interior composition of amino acids in extracellular proteins of mesophiles	2	NS	NS	NS
FUKS010108	Interior composition of amino acids in nuclear proteins	2	NS	NS	NS
FUKS010109	Entire chain composition of amino acids in intracellular proteins of	2	NS	NS	NS

FUKS010110	Entire chain composition of amino acids in intracellular proteins of	2	NS	NS	NS
FUKS010111	Entire chain composition of amino acids in extracellular proteins of	2	NS	NS	NS
FUKS010112	Entire chain compositino of amino acids in nuclear proteins	2	NS	NS	NS
MITS020101	Amphiphilicity index	2	0.0375765	NS	NS
TSAJ990101	Volumes including the crystallographic waters using the ProtOr	2	NS	NS	NS
TSAJ990102	Volumes not including the crystallographic waters using the ProtOr	2	NS	NS	NS
COSI940101	Electron-ion interaction potential values	2	NS	NS	NS
PONP930101	Hydrophobicity scales	2	0.018213073	NS	NS
WILM950101	Hydrophobicity coefficient in RP-HPLC, C18 with 0.1%TFA/MeCN/H2O	2	NS	NS	NS
WILM950102	Hydrophobicity coefficient in RP-HPLC, C8 with 0.1%TFA/MeCN/H2O	2	NS	NS	NS
WILM950103	Hydrophobicity coefficient in RP-HPLC, C4 with 0.1%TFA/MeCN/H2O	2	NS	NS	NS
WILM950104	Hydrophobicity coefficient in RP-HPLC, C18 with 0.1%TFA/2-PrOH/MeCN/H2O	2	NS	NS	NS
KUHL950101	Hydrophilicity scale	2	NS	NS	NS
GUOD860101	Retention coefficient at pH 2	2	0.006778846	0.017559923	NS
JURD980101	Modified Kyte-Doolittle hydrophobicity scale	2	0.0257184	NS	NS
BASU050101	Interactivity scale obtained from the contact matrix	2	0.029995038	NS	NS
BASU050102	Interactivity scale obtained by maximizing the mean of correlation	2	0.006959547	0.017559923	NS
BASU050103	Interactivity scale obtained by maximizing the mean of correlation	2	0.031547444	NS	NS
SUYM030101	Linker propensity index	2	0.032712	NS	NS
PUNT030101	Knowledge-based membrane-propensity scale from 1D_Helix in MPtopo databases	2	0.040020885	NS	NS
PUNT030102	Knowledge-based membrane-propensity scale from 3D_Helix in MPtopo databases	2	NS	NS	NS
GEOR030101	Linker propensity from all dataset	2	NS	NS	NS
GEOR030102	Linker propensity from 1-linker dataset	2	NS	NS	NS
GEOR030103	Linker propensity from 2-linker dataset	2	NS	NS	NS
GEOR030104	Linker propensity from 3-linker dataset	2	NS	NS	NS
GEOR030105	Linker propensity from small dataset	2	0.001692	0.004841	0.013739667
GEOR030106	Linker propensity from medium dataset	2	NS	NS	NS
GEOR030107	Linker propensity from long dataset	2	NS	NS	NS
GEOR030108	Linker propensity from helical	2	NS	NS	NS
GEOR030109	Linker propensity from non-helical	2	NS	NS	NS
ZHOH040101	The stability scale from the knowledge-based atom-atom potential	2	0.0034075	0.0073555	NS
ZHOH040102	The relative stability scale extracted from mutation experiments	2	0.035541479	NS	NS
ZHOH040103	Buriability	2	0.0034075	0.0073555	0.007687111
BAEK050101	Linker index	2	0.028853347	NS	NS

HARY940101	Mean volumes of residues buried in protein interiors	2	NS	NS	NS
PONJ960101	Average volumes of residues	2	NS	NS	NS
DIGM050101	Hydrostatic pressure asymmetry index, PAI	2	NS	NS	NS
WOLR790101	Hydrophobicity index	2	NS	NS	NS
OLSK800101	Average internal preferences	2	0.026220245	NS	NS
KIDA850101	Hydrophobicity-related index	2	NS	NS	NS
GUYH850102	Apparent partition energies calculated from Wertz-Scheraga index	2	0.003600923	0.006916421	0.005943692
GUYH850104	Apparent partition energies calculated from Janin index	2	NS	NS	NS
GUYH850105	Apparent partition energies calculated from Chothia index	2	NS	NS	NS
JACR890101	Weights from the IFH scale	2	NS	NS	NS
COWR900101	Hydrophobicity index, 3.0 pH	2	0.023778968	NS	NS
BLAS910101	Scaled side chain hydrophobicity values	2	0.033492536	NS	NS
CASG920101	Hydrophobicity scale from native protein structures	2	0.005276129	0.009724138	0.0021996
CORJ870101	NNEIG index	2	0.005584976	0.017559923	0.019896
CORJ870102	SWEIG index	2	0.001692	0.004841	0.010416375
CORJ870103	PRIFT index	2	0.005909182	0.01491161	NS
CORJ870104	PRILS index	2	0.00777931	0.020794033	NS
CORJ870105	ALTFT index	2	0.007342071	0.030616054	NS
CORJ870106	ALTLS index	2	0.008687419	0.0343664	NS
CORJ870107	TOTFT index	2	0.005584976	0.017559923	NS
CORJ870108	TOTLS index	2	0.005584976	0.017559923	NS
MIYS990101	Relative partition energies derived by the Bethe approximation	2	0.005584976	0.017559923	NS
MIYS990102	Optimized relative partition energies - method A	2	0.005584976	0.017559923	NS
MIYS990103	Optimized relative partition energies - method B	2	0.003276571	0.007770667	0.00516624
MIYS990104	Optimized relative partition energies - method C	2	0.0018048	0.004902462	0.002417143
MIYS990105	Optimized relative partition energies - method D	2	0.001692	0.003321333	0.0021996
ENGD860101	Hydrophobicity index	2	NS	NS	NS
FASG890101	Hydrophobicity index	2	0.012133833	0.025372056	0.035189176
KARS160101	Number of vertices	2	NS	NS	NS
KARS160102	Number of edges	2	NS	NS	NS
KARS160103	Total weighted degree of the graph	2	NS	NS	NS
KARS160104	Weighted domination number	2	NS	NS	NS
KARS160105	Average eccentricity	2	NS	NS	NS
KARS160106	Radius	2	NS	NS	NS

KARS160107	Diameter	2	NS	NS	NS
KARS160108	Average weighted degree	2	NS	NS	NS
KARS160109	Maximum eigenvalue of the weighted Laplacian matrix of the graph	2	NS	NS	NS
KARS160110	Minimum eigenvalue of the weighted Laplacian matrix of the graph	2	NS	NS	NS
KARS160111	Average eigenvalue of the Laplacian matrix of the the graph	2	NS	NS	NS
KARS160112	Second smallest eigenvalue of the Laplacian matrix of the graph	2	NS	NS	NS
KARS160113	Weighted domination number using the atomic number	2	NS	NS	NS
KARS160114	Average weighted eccentricity based on the the atomic number	2	NS	NS	NS
KARS160115	Weighted radius based on the atomic number	2	NS	NS	NS
KARS160116	Weighted diameter based on the atomic number	2	NS	NS	NS
KARS160117	Total weighted atomic number of the graph	2	NS	NS	NS
KARS160118	Average weighted atomic number or degree based on atomic number in the graph	2	NS	NS	NS
KARS160119	Weighted maximum eigenvalue based on the atomic numbers	2	NS	NS	NS
KARS160120	Weighted minimum eigenvalue based on the atomic numbers	2	NS	NS	NS
KARS160121	Weighted average eigenvalue based on the atomic numbers	2	NS	NS	NS
KARS160122	Weighted second smallest eigenvalue of the weighted Laplacian matrix	2	NS	NS	NS
poly201301	GUA-affinity	3	0.027083394	0.047258242	0.003791333
poly201302	CYT-affinity	3	NS	NS	NS
poly201303	URA-affinity	3	NS	NS	NS
poly201304	ADE-affinity	3	0.010331826	0.022122269	NS
poly201305	PUR-affinity	3	0.019784864	0.030616054	0.00516624
poly201306	PYR-affinity	3	0.012500712	0.018707593	0.003791333

1. Atchley, W. R., et al. (2005) Proc Natl Acad Sci USA 102:6395-6400.
2. Kawashima S., et al. (2008) Nucleic Acids Res 36:D202-205.
3. Polyansky A. A., Zagrovic, B. (2013) Nucleic Acids Res 41:8434-8443.

Table S2. Codon position-specific patterns of translational errors in *E. coli*

	First position	Second position	Third position
Relative frequency of error	1.03	1	1.50
Transition/transversion rate ratio of error	1.75	1.29	1.52

Table S3. Controlling MS1 explains MS3 of the SGC. The adjusted *P*-value shows the significance level of the SGC's frameshift-robustness after the control of mismatch-robustness in the same property.

Amino acid property ID	Adjusted *P*-value
OOBM770103	0.666666667
NISK860101	0.193427849
GEOR030105	0.193427849
NOZY710101	0.172405215
MIYS850101	0.169165705
FASG890101	0.157044458
MEIH800102	0.149122486
NISK800101	0.138529303
SWER830101	0.138529303
CORJ870101	0.138529303
CORJ870102	0.138529303
MEIH800103	0.138529303
PARS000102	0.138529303
RACS820105	0.137137287
PONP800102	0.136722268
WOEC730101	0.136450381
DESM900102	0.120766256
GRAR740102	0.111336033
NAKH900108	0.111336033
NADH010102	0.111336033
WERD780101	0.111336033
BIOV880101	0.111336033
MIYS990103	0.111336033
VINM940104	0.111336033
LEVM760101	0.111336033
ZHOH040103	0.111336033
BIOV880102	0.111336033
FAUJ830101	0.111336033
RADA880108	0.111336033
WIMW960101	0.111336033
GUYH850102	0.111336033
RACS820103	0.111336033
DESM900101	0.111336033
NADH010104	0.111336033
KARP850102	0.111336033
NADH010103	0.111336033
FUKS010102	0.111336033
VINM940101	0.111336033
PONP800103	0.111336033

KRIW710101	0.111336033
MIYS990105	0.111336033
KRIW790101	0.111336033
HOPT810101	0.111336033
FUKS010101	0.111336033
KARP850103	0.111336033
ROSG850102	0.111336033
RACS770103	0.111336033
poly201306	0.111336033
poly201305	0.111336033
MIYS990104	0.111336033
CASG920101	0.111336033
poly201301	0.111336033
VINM940103	0.111336033
KRIW790102	0.111336033
FUKS010104	0*

* See main text

Table S4. Controlling MS2 explains MS3 of the SGC. The adjusted *P*-value shows the significance level of the SGC's frameshift-robustness after the control of mismatch-robustness in the same property.

Amino acid property ID	Adjusted *P*-value
GEOR030105	0.205882353
GRAR740102	0.203773585
FASG890101	0.141456026
NISK800101	0.138629014
MEIH800103	0.124615384
NISK860101	0.124615384
CORJ870101	0.122950819
PARS000102	0.118691753
PONP800102	0.118691753
RACS820105	0.118691753
NOZY710101	0.115281979
WOEC730101	0.114164905
LEVM760101	0.105659362
SWER830101	0.10397946
VINM940101	0.10397946
CORJ870102	0.10397946
MEIH800102	0.10397946
DESM900102	0.10397946
NADH010102	0.10397946
VINM940104	0.10397946
FAUJ830101	0.098039216
BIOV880102	0.096256684
NAKH900108	0.090938145
RACS820103	0.090938145
DESM900101	0.087526427
KARP850102	0.087075168
KRIW710101	0.086559321
HOPT810101	0.086559321
GUYH850102	0.086559321
RADA880108	0.084705882
NADH010103	0.083571428
MIYS850101	0.083571428
NADH010104	0.083571428
KARP850103	0.083571428
ZHOH040103	0.083571428
BIOV880101	0.083571428
FUKS010101	0.083571428

KRIW790101	0.080929187
poly201305	0.080929187
poly201306	0.077873918
PONP800103	0.077873918
MIYS990103	0.077873918
poly201301	0.066259994
RACS770103	0.066259994
VINM940103	0.058909091
CASG920101	0.058909091
KRIW790102	0.039448053
FUKS010104	0
WIMW960101	0
FUKS010102	0
WERD780101	0
MIYS990104	0
MIYS990105	0
ROSG850102	0

Legends of supplementary figures

Fig. S1. Correlation between mismatch- and frameshift-robustness in fake amino acid properties simulated using a normal distribution. Data shown are based on 10,000 RGCs. (**A**) Frequency distribution of the across-RGC correlation between frameshift-robustness (MS3) and mismatch-robustness (MS1 or MS2) among 564 fake amino acid properties. (**B-C**) Frequency distribution of the across-RGC correlation between frameshift-robustness (MS3) and mismatch-robustness measured by MS1 (**B**) or MS2 (**C**) among the 564 fake amino acid properties. In (B) and (C), MS3 is calculated based on all frameshift events (blue), mismatch-like frameshift events (orange), or mismatch-unlike frameshift events (green). The correlation between mismatch-robustness (MS1 or MS2) and mismatch-like frameshift-robustness (MS3) differs significantly from that between mismatch-robustness and mismatch-unlike frameshift-robustness ($P < 10^{-93}$, Wilcoxon signed-rank test) in both (B) and (C).

Fig. S2. Correlation between MS1 and MS3 across the 55 amino acid properties that show significant frameshift-robustness in the SGC. The four properties with the highest correlations with MS3 of FUKS010104 (indicated by green color) are marked in red.

Fig. S3. Correlation between MS2 and MS3 across the 55 amino acid properties that show significant frameshift-robustness in the SGC.

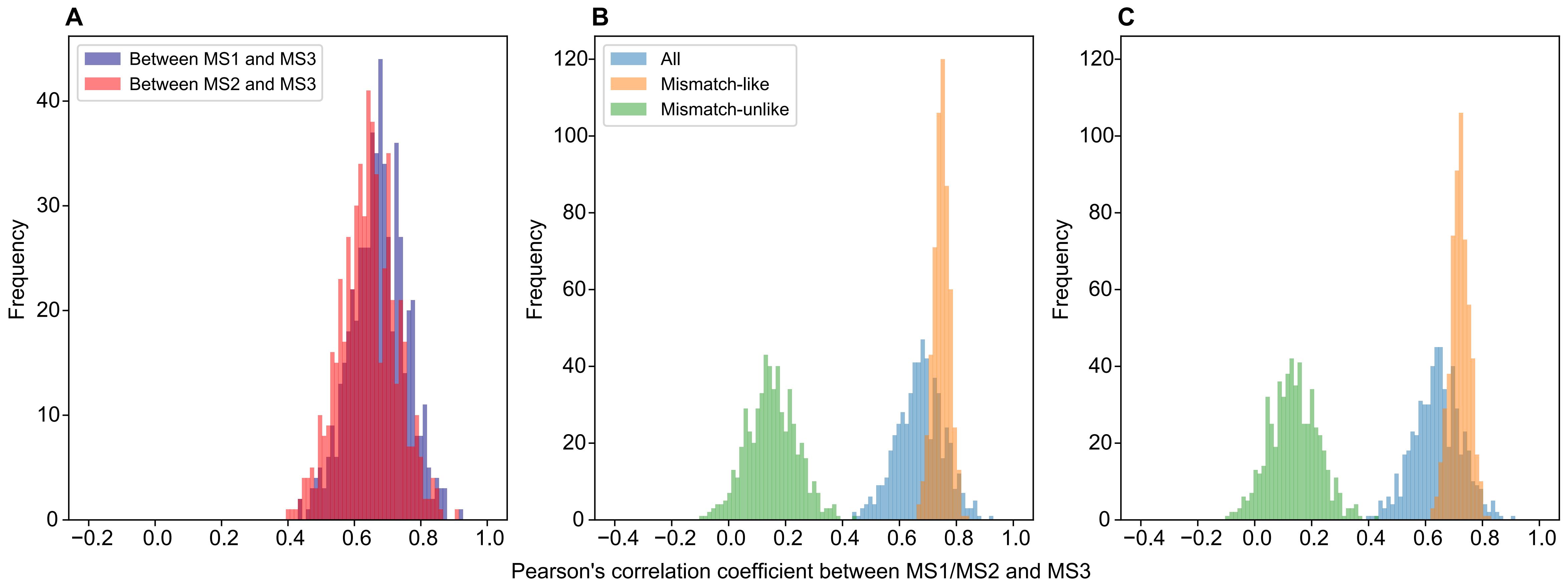
A
Between MS1 and MS3
Between MS2 and MS3
Frequency
B
All
Mismatch-like
Mismatch-unlike
Frequency
C
Frequency
Pearson's correlation coefficient between MS1/MS2 and MS3

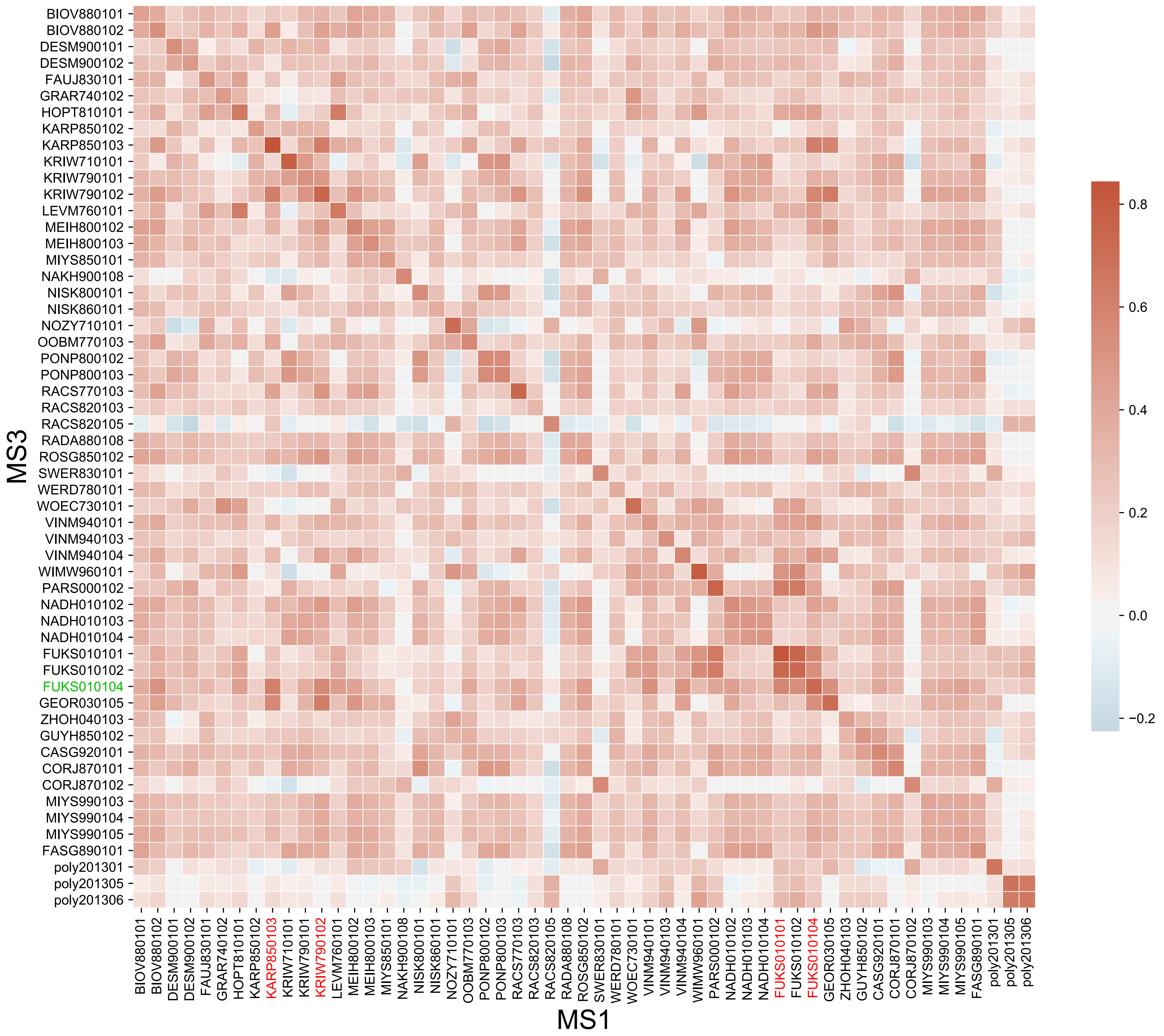
MS3
MS1
BIOV880101
BIOV880102
DESM900101
DESM900102
FAUJ830101
GRAR740102
HOPT810101
KARP850102
KARP850103
KRIW710101
KRIW790101
KRIW790102
LEVM760101
MEIH800102
MEIH800103
MIYS850101
NAKH900108
NISK800101
NISK860101
NOZY710101
OOBM770103
PONP800102
PONP800103
RACS770103
RACS820103
RACS820105
RADA880108
ROSG850102
SWER830101
WERD780101
WOEC730101
VINM940101
VINM940103
VINM940104
WIMW960101
PARS000102
NADH010102
NADH010103
NADH010104
FUKS010101
FUKS010102
FUKS010104
GEOR030105
ZHOH040103
GUYH850102
CASG920101
CORJ870101
CORJ870102
MIYS990103
MIYS990104
MIYS990105
FASG890101
poly201301
poly201305
poly201306
0.8
0.6
0.4
0.2
0.0
−0.2

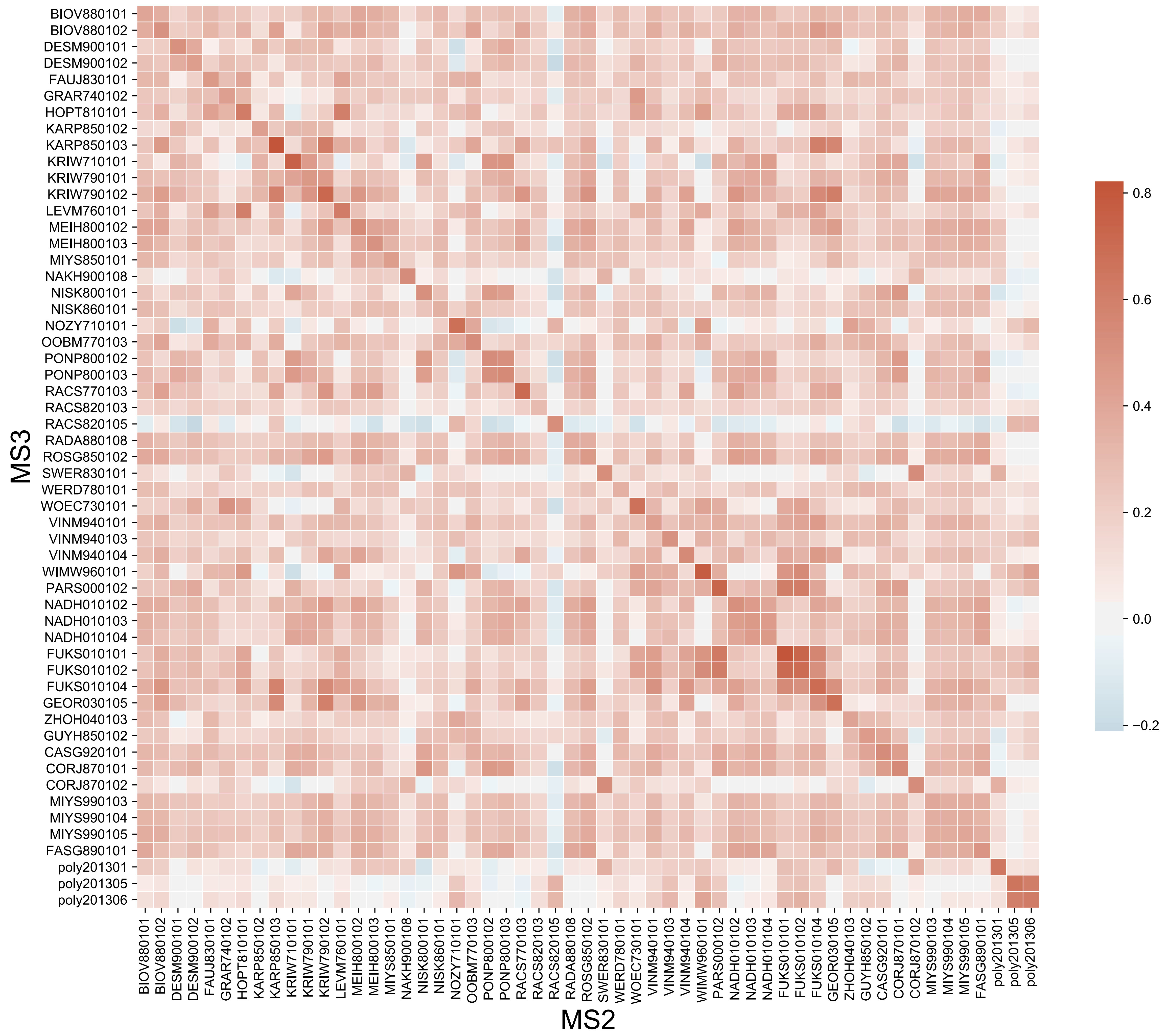

MS3
MS2
BIOV880101
BIOV880102
DESM900101
DESM900102
FAUJ830101
GRAR740102
HOPT810101
KARP850102
KARP850103
KRIW710101
KRIW790101
KRIW790102
LEVM760101
MEIH800102
MEIH800103
MIYS850101
NAKH900108
NISK800101
NISK860101
NOZY710101
OOBM770103
PONP800102
PONP800103
RACS770103
RACS820103
RACS820105
RADA880108
ROSG850102
SWER830101
WERD780101
WOEC730101
VINM940101
VINM940103
VINM940104
WIMW960101
PARS000102
NADH010102
NADH010103
NADH010104
FUKS010101
FUKS010102
FUKS010104
GEOR030105
ZHOH040103
GUYH850102
CASG920101
CORJ870101
CORJ870102
MIYS990103
MIYS990104
MIYS990105
FASG890101
poly201301
poly201305
poly201306
0.8
0.6
0.4
0.2
0.0
−0.2
